# Maternal pre-pregnancy obesity and health care utilization and costs in the offspring

**DOI:** 10.1038/s41366-018-0149-3

**Published:** 2018-07-13

**Authors:** Stefan Kuhle, Adam Muir, Christy G. Woolcott, Mary M. Brown, Sarah D. McDonald, Mohamed Abdolell, Linda Dodds

**Affiliations:** 1 Departments of Obstetrics & Gynaecology and Pediatrics, Perinatal Epidemiology Research Unit, Dalhousie University, Halifax, NS Canada; 2 Department of Obstetrics & Gynecology, Division of Maternal-Fetal Medicine, McMaster University, Hamilton, ON Canada; 3 Department of Radiology, McMaster University, Hamilton, ON Canada; 4 Department of Research Methods, Evidence and Impact, McMaster University, Hamilton, ON Canada; 5 Department of Diagnostic Radiology, Dalhousie University, Halifax, NS Canada

**Keywords:** Paediatrics, Epidemiology

## Abstract

**Background/objective:**

The association between maternal pre-pregnancy obesity and adverse child health outcomes is well described, but there are few data on the relationship with offspring health service use. We examined the influence of maternal pre-pregnancy obesity on offspring health care utilization and costs over the first 18 years of life.

**Methods:**

This was a population-based retrospective cohort study of children (*n* = 35,090) born between 1989 and 1993 and their mothers, who were identified using the Nova Scotia Atlee Perinatal Database and linked to provincial administrative health data from birth through 2014. The primary outcome was health care utilization as determined by the number and cost of physician visits, hospital admissions and days, and high utilizer status (>95th percentile of physician visits). The secondary outcome was health care utilization by ICD chapter. Maternal pre-pregnancy weight was categorized as normal weight, overweight, or obese. Multivariable-adjusted regression models were used to examine the association between maternal weight status and offspring health care use.

**Results:**

Children of mothers with pre-pregnancy obesity had more physician visits (10%), hospital admissions (16%), and hospital days (10%) than children from mothers of normal weight over the first 18 years of life. Offspring of mothers with obesity had C$356 higher physician costs and C$1415 hospital costs over 18 years than offspring of normal weight mothers. Children of mothers with obesity were 1.74 times more likely to be a high utilizer of health care and had higher rates of physician visits and hospital stays for nervous system and sense organ disorders, respiratory disorders, and gastrointestinal disorders compared to children of normal weight mothers.

**Conclusion:**

Our findings suggest that maternal pre-pregnancy overweight and obesity are associated with slightly higher offspring health care utilization and costs in the first 18 years of life.

## Introduction

Obesity is considered a significant public health concern in high income countries. In Canada, 13% of women of childbearing age are obese, with the highest rates for obesity occurring in the Atlantic provinces at 24% [1]. Maternal obesity is associated with pregnancy, delivery, and neonatal complications [2–4]. As a result, the costs of prenatal care and hospital stays associated with delivery for mothers with obesity are significantly higher than those for normal weight mothers [5–7].

Excess maternal weight has also consistently been identified as a risk factor for childhood overweight and obesity [3]. Childhood obesity in turn is associated with a broad range of health conditions [8] including asthma and other respiratory disorders [9, 10], sleep apnea [11], metabolic syndrome and type 2 diabetes [12, 13], and mental health problems [14]. Maternal obesity, therefore, may be related these childhood conditions through mediation by birth weight or offspring body mass index (BMI) in childhood. In keeping with this hypothesis, associations between maternal obesity and childhood health outcomes such as asthma [15–17], metabolic syndrome [18, 19], and mental health conditions [20, 21] have been reported.

Despite the strong evidence for the association between maternal obesity and offspring obesity and other adverse conditions, there is limited information on the intergenerational burden of maternal obesity on offspring health care use and costs. Three studies have suggested that maternal pre-pregnancy obesity is positively associated with both offspring health care utilization and cost in the short term [22–24]. However, the long-term health care utilization of children born to mothers with obesity has not been examined yet in a population-based study. Therefore, the objective of this study was to investigate the relationship between pre-pregnancy weight status and offspring health care utilization and costs in childhood through young adulthood in a population-based cohort from Nova Scotia, Canada.

## Material and methods

### Study design and setting

The current study is a population-based retrospective cohort study in the Canadian province of Nova Scotia. All live births without major congenital malformations to mothers resident in Nova Scotia between 1989 and 1993 recorded in the Nova Scotia Atlee Perinatal Database (NSAPD) were included and followed until age 18 years. Nova Scotia has a population of approximately 950,000 as of 2015 with a median age of 44 years [25]; the median income was just under C$29,000 annually in 2013 [26]. All Canadian provinces have a single-payer universal health care system that pays for essential health services for all residents.

The study was approved by the IWK Health Centre Research Ethics Board (File # 1015756), the Joint Data Access Committee, and the Health Data Nova Scotia Data Access Committee.

### Data sources

#### NOVA SCOTIA ATLEE PERINATAL DATABASE

The NSAPD contains information on all pregnancies and births to mothers resident in Nova Scotia since 1988, including demographics, diagnoses, morbidity, and mortality data for both mother and infant, for each pregnancy. Information is obtained from standard provincial antenatal and hospital data collection forms by health records personnel. The quality and validity of the data entered into NSAPD are ensured through regular data checks and validation studies [27].

#### Administrative health data

Administrative health databases that were used in the current study included the physician billings database, the hospital discharge abstracts databases, and the Insured Patient Registry. The Medical Services Insurance physician billings database collected administrative records encompassing diagnostic and procedure codes for each insured service rendered by a physician that was compensated by the Nova Scotia health care system since 1989; for each service rendered, the database contains diagnostic codes in the International Classification of Disease version 9 (ICD-9) format (one from 1989 to 1996, up to three after 1996) and procedure codes. Information on hospital admissions was collected in the Admissions/Separations/Day Surgery hospital database (1989–1994) and the Canadian Institute of Health Information Discharge Abstract Database (since 1995). Information in these databases includes patient demographics, diagnostic and procedure codes, and specialty services received. Diagnostic codes are in ICD-9 format from 1989–2000 and ICD-10CA format from 2001 forward. The Insured Patients Registry holds longitudinal information about each beneficiary of Nova Scotia health care services; the registry was used to identify children in the cohort who left the province or died during the study period.

#### Data linkage

Information from the NSAPD was linked with administrative health data by Health Data Nova Scotia using deterministic matching by health card number. In total, there were 48,790 eligible live births between 1989 and 1993. After removing observations with missing or implausible values for birth weight for gestational age (|*z*-score| > 5) [28], 47,450 births remained. Of these, 42,999 (91%) offspring from 35,285 mothers could successfully be linked with their information in the administrative databases.

### Outcomes

The primary outcome was health care utilization and costs in the offspring during the first 18 years of life, as determined by the number of physician visits, number of hospital days, physician and hospital costs, and high utilizer status (defined as being above the 95th percentile of the total number of physician visits). Secondary outcomes were health care utilization and cost by disease groups based on the ICD-9 chapters. Physician and hospital costs are presented in 2014 Canadian Dollars as adjusted by the Canadian Consumer Price Index [29]. Since Canadian hospitals do not operate on a fee-for-service basis, we were unable to directly determine the cost of a hospital day from our data. We therefore estimated the cost per hospital day as C$1221 from the average cost of a hospital stay of C$6107 and average length of stay of 5 days in Nova Scotia in 2014–2015.

### Exposure

The main exposure was maternal pre-pregnancy weight status (normal weight [<25 kg/m^2^], overweight [25 to <30 kg/m^2^], and obese [≥30 kg/m^2^] [30]); underweight women were included in the normal weight group. Pre-pregnancy BMI was calculated from height and weight information collected by self-report at the first prenatal visit. Maternal height has been routinely collected for the NSAPD since 2003 and has been backfilled where available for women with pregnancies before that year. For mothers with missing height information, we estimated maternal weight cutpoints that best predict maternal weight status based on data from 77 297 mothers with complete weight and height information in the NSAPD between 2003 and 2015 (normal weight: <68.0 kg; overweight: 68.0–76.6 kg; obese: ≥76.7 kg). These weight-based categories have a high sensitivity (89–96%) and specificity (90–95%) with the highest values observed for the obese category. When regressing common perinatal outcomes (gestational diabetes, mode of delivery, 5-min Apgar score <7, birth weight for gestational age, pre-eclampsia) on imputed and recorded weight status, effect estimates from the corresponding models did not differ by more than 10% and the imputation-based estimates were generally closer to the null than the complete case estimates (Woolcott and Kuhle, unpublished). These findings confirm the accuracy and validity of the body weight-based categories.

### Confounders

**Fig. 1:**
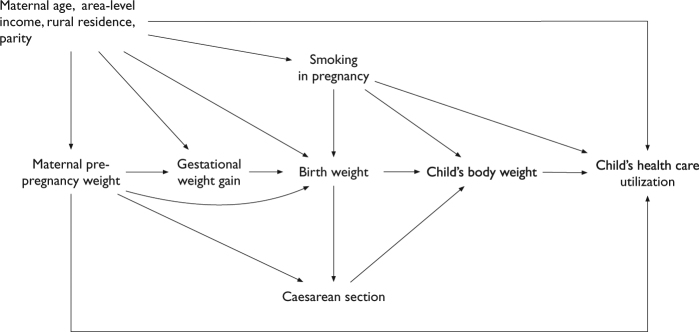
Directed acyclic graph representing the effect of maternal pre-pregnancy weight on offspring health care utilization

Income quintile at the dissemination (neighbourhood) area-level determined from Census of Canada information, maternal age at birth (years), residence at birth (urban or rural, based on postal code), parity (1, 2, and ≥3), and smoking during pregnancy (any smoking reported at either the first prenatal assessment or upon admission to the labour ward) were identified as confounders using a directed acyclic graph (Fig. 1).

### Statistical analysis

Descriptive statistics were summarized by maternal pre-pregnancy weight status. The association between pre-pregnancy weight status and the number of physician visits of the offspring was estimated using a generalized linear model with a negative binomial distribution and a log link function. The relationship between maternal weight status and the number of hospital admissions was examined using a two-part hurdle model (logistic regression to model having ever stayed in a hospital, and zero-truncated negative binomial regression to model the rates of hospital admissions and hospital days). Negative binomial regression was used to model the relationship between maternal weight status and the number of hospital days. Results for the negative binomial models are presented as incidence rate ratios (IRR) with 95% confidence intervals (CI). A generalized linear model with a gamma distribution and a log link function was used to estimate the association between maternal weight status and total physician costs. Results are presented as multiplicative changes in the expected costs (cost ratios) relative to offspring of normal weight mothers. The relationship between maternal pre-pregnancy weight status and high utilizer status was examined using logistic regression. Results are expressed as odds ratios (OR) with 95% CI. All models were adjusted for maternal smoking, maternal age, parity, area-level income, and area of residence. Using all confounders and outcome variables, multiple imputation (*n* = 5) with chained equations was executed (25 iterations) to impute missing values of the model covariates [31]. An ordered logit model was used to impute area-level income and parity, whereas a logistic regression was used to impute smoking status during pregnancy.

We estimated the difference in physician and hospital costs over the first 18 years of life between children of mothers with overweight and obesity, respectively, and children of normal weight mothers from the corresponding regression models.

To explore how the associations change over age, the IRRs for physician visits and hospital admissions were estimated monthly up to 18 years. Estimates were fit with a smoothed, adjusted generalized additive model and plotted by maternal pre-pregnancy weight status.

Rates of physician visits and hospital admissions over the first 18 years of life were calculated by pre-pregnancy weight status for ICD chapter-based disease groups.

The statistical software package R with user packages *MASS* [32], *pscl* [33], *ggplot2* [34], and *mgcv* [35] was used for all analyses.

## Results

After removing children for whom maternal weight information was unavailable (*n* = 3535) or who did not live in the province for the full 18 years (*n* = 4374), the final analysis sample included 35,090 children delivered by 29,392 mothers in Nova Scotia between 1989 and 1993. Descriptive characteristics of the sample are summarized by maternal pre-pregnancy weight status in Table 1. 16 and 15% of children were born to mothers with overweight and obesity, respectively. Most children were born to mothers residing in an urban area, and almost 30% of children were born to mothers who smoked during pregnancy. Offspring of mothers with obesity were more likely to reside in less affluent neighbourhoods and in a rural area than offspring of normal weight mothers. Maternal weight status groups were comparable with regard to maternal age, offspring sex, and smoking during pregnancy. Children with missing maternal weight or who did not live the full 18 years in the province (*n* = 7909) were slightly less likely to come from the most affluent neighbourhoods (14 vs. 16%), to live in a rural area (39 vs. 43%), and to have only one child (42 vs. 45%), and tended to have younger mothers (27.0 vs. 27.4 years) than those who were included in the analysis.

**Table 1:** Sample characteristics by maternal pre-pregnancy weight status (*n* = 35,090)

	Normal weight 69.4% *n* = 24 341	Overweight 15.8% *n* = 5556	Obese 14.8% *n* = 5193
Maternal age [years] (mean, SD)	27.3 (5.1)	27.6 (5.0)	27.5 (4.8)
*Area-level income quintile (%)*			
Q1 (lowest)	19.1	20.1	21.9
Q2	21.6	22.4	23.4
Q3	20.9	21.1	21.5
Q4	20.0	20.0	18.6
Q5 (highest)	17.1	15.2	13.3
Missing	1.3	1.2	1.4
*Area of residence (%)*			
Urban	58.8	56.3	52.2
Rural	41.2	43.7	47.8
*Parity (%)*			
1	45.9	42.4	41.3
2	35.1	35.8	36.6
**≥**3	19.0	21.9	22.1
*Smoking during pregnancy (%)*			
No	67.2	68.1	67.9
Yes	29.6	28.7	28.9
Missing	4.5	4.5	4.4
*Offspring sex (%)*			
Female	49.1	50.3	49.5
Male	50.9	49.7	50.5

*SD* standard deviation

**Fig. 2:**
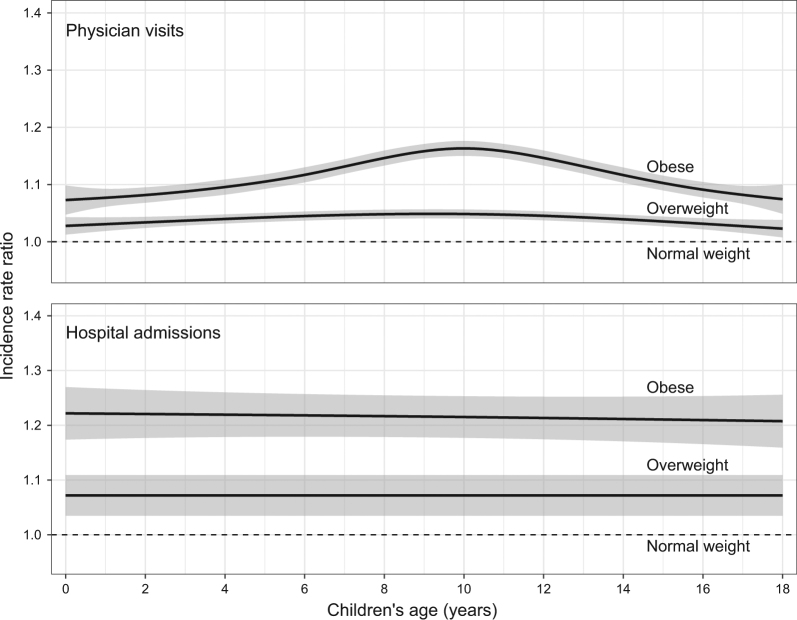
Smoothed incidence rate ratios with 95% confidence intervals of offspring physician visits (top) and hospital admissions (bottom) over the first 18 years of life by maternal pre-pregnancy weight status (relative to normal weight)

In the adjusted regression models (Table 2), maternal pre-pregnancy weight status was associated with offspring health care utilization and costs in the first 18 years of life. Compared to children of normal weight mothers, children of mothers with overweight or obesity had 4 and 10% more physician visits, respectively. The difference in the rate of physician visits for children of mothers with obesity relative to those of normal weight mothers peaked at 10 years (IRR 1.17, Fig. 2), while the relative rates of hospital admissions remained constant over age in the three groups. Children of mothers with obesity had more hospital admissions (16%), longer hospital stays (10%) and were 74% more likely to be high utilizers of physician services than children of normal weight mothers. Physician costs were also higher in both children of mothers with overweight (3%) and obesity (9%) compared to children from normal weight mothers, corresponding to C$113 (95% CI 37, 191) and C$356 (95% CI 275, 438) adjusted excess physician costs during the first 18 years of life. We estimated the adjusted hospital cost differences over the first 18 years of life among children of mothers with overweight or obesity relative to offspring of normal weight mothers to be C$231 (95% CI -403, 847) and C$1415 (95% CI 590, 2285), respectively.

**Table 2:** Median and mean, and unadjusted and adjusted incidence rate ratios (number of physician visits, number of hospital days), cost ratios (physician costs), and odds ratios (high utilizers) with 95% confidence intervals for the association between maternal pre-pregnancy weight status and health care utilization in the offspring

	Maternal pre-pregnancy weight status		
	Normal weight	Overweight	Obese
*Number of physician visits*			
Median (IQR)	78 (55–109)	81 (56-112)	84 (59-118)
Mean (SD)	87.2 (48.0)	89.9 (52.8)	95.3 (53.3)
Unadjusted IRR (95% CI)	1.00 (ref)	1.03 (1.02, 1.05)	1.09 (1.07, 1.11)
Adjusted IRR (95% CI)	1.00 (ref)	1.04 (1.03, 1.05)	1.10 (1.08, 1.12)
*Number of hospital admissions*			
Median (IQR)	2 (1–3)	2 (1–3)	2 (1–3)
Mean (SD)	2.3 (2.0)	2.4 (2.7)	2.6 (2.4)
Unadjusted IRR (95% CI)	1.00 (ref)	1.08 (1.00, 1.17)	1.16 (1.09, 1.23)
Adjusted IRR (95% CI)	1.00 (ref)	1.08 (1.00, 1.16)	1.16 (1.09, 1.23)
*Number of hospital days*			
Median (IQR)	6 (5–10)	7 (5–10)	7 (5–11)
Mean (SD)	9.8 (16.0)	9.9 (25.3)	10.8 (17.0)
Unadjusted IRR (95% CI)	1.00 (ref)	1.02 (0.99, 1.04)	1.10 (1.07, 1.13)
Adjusted IRR (95% CI)	1.00 (ref)	1.02 (0.99, 1.04)	1.10 (1.06, 1.13)
*Physician costs*			
Median (IQR)	C$3101 (2120–4492)	C$3167 (2165–4608)	C$3350 (2256–4948)
Mean (SD)	3615 (2370)	3701 (2510)	3953 (2613)
Unadjusted cost ratio (95% CI)	1.00 (ref)	1.02 (1.00, 1.05)	1.09 (1.06, 1.11)
Adjusted cost ratio (95% CI)	1.00 (ref)	1.03 (1.01, 1.05)	1.09 (1.07, 1.12)
*High utilizer (Physician visits)*			
%	4.4	5.4	7.4
Unadjusted OR (95% CI)	1.00 (ref)	1.26 (1.11, 1.43)	1.71 (1.52, 1.93)
Adjusted OR (95% CI)	1.00 (ref)	1.28 (1.13, 1.45)	1.74 (1.54, 1.96)

Models were adjusted for maternal age, parity, area-level income, area of residence, and maternal smoking during pregnancy

*CI* confidence interval, *IQR* interquartile range, *IRR* incidence rate ratio, *OR* odds ratio, *ref* reference, *SD* standard deviation

**Fig. 3:**
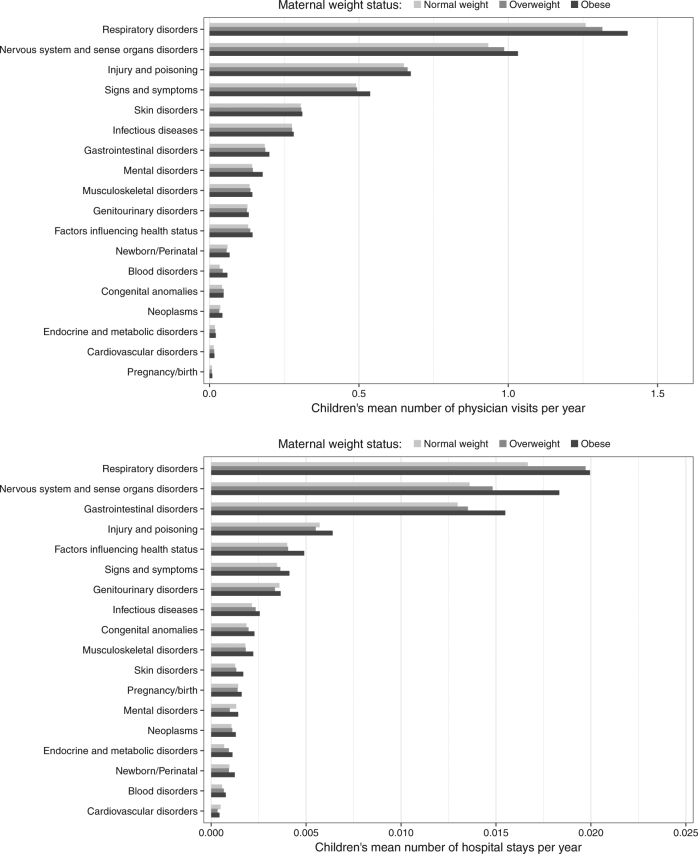
Children’s mean number of physician visits (top) and hospital admissions (bottom) per year over the first 18 years of life, by ICD chapter and maternal pre-pregnancy weight status

Rates of health care utilization by ICD chapter of the primary diagnosis for each visit are shown in Fig. 3. Children of mothers with obesity had higher rates of physician visits for most categories compared to children of normal weight mothers. For hospital admissions, distinctly higher rates in offspring of mothers with obesity compared to offspring of normal weight mothers were observed for nervous system and sense organ disorders, respiratory disorders, and gastrointestinal disorders (Fig. 3).

## Discussion

Our analysis demonstrates a higher rate of use and costs of health care over the first 18 years in the offspring of mothers with obesity compared to offspring of normal weight mothers. For physician visits, the difference in rate of use was 10%, which resulted in an increase in associated costs of C$356. The rate difference for hospital days was similar and corresponded to an estimated C$1415 difference in costs. Taken together, children of mothers with obesity incur approximately $1770 higher direct health care costs in the first 18 years of life than children of mothers of normal weight.

The difference in the rate of physician visits between children of mothers with obesity and children of mothers of normal weight was comparable to the findings from a recent retrospective study of 609 children in the UK, which found a 13% difference in physician visits over the first year of life [22]. The same study also reported a 39% higher rate of hospital admissions for offspring of mothers with obesity compared to offspring of normal weight mothers, which is twice the difference that we observed (data not shown) [22]. A recent five-year prospective birth cohort study that used administrative data from 2800 children admitted to an Australian hospital reported a 48% higher rate of admissions for children of mothers with obesity compared to children of normal weight mothers [23]. Our finding that the offspring of mothers with obesity are about 1.7 times more likely to be above the 95th percentile for physician visits than offspring of normal weight mothers indicates that the former may be more likely to suffer from chronic health conditions that require frequent physician visits compared to offspring of mothers of normal weight.

Our findings demonstrate that maternal obesity is associated with risks for the mother but also for their offspring, which is in keeping with other studies in this area [22, 23]. The excess health care cost differences for offspring of obese mothers in our study may be a precursor for more complex, and chronic health conditions, leading to a far larger disease and cost burden in adulthood. The findings of our study may be explained by children of mothers with obesity developing obesity themselves as a result of the “intergenerational vicious cycle” [36] that perpetuates the obesity epidemic. We were unable to test this hypothesis as BMI is not captured in administrative health data in Canada. Another possible explanation is that birth by Caesarean section, which is more frequently seen in mothers with obesity [37], led to an increased number of microbiome-related chronic conditions such as asthma, allergies, or diabetes [38]. Lastly, it is possible that our findings are unrelated to the intergenerational transference of obesity or the microbiome but are due to unmeasured confounding by socioeconomic status and associated adverse health behaviours.

Our finding of higher rates of physician visits and hospital admissions for respiratory, nervous system, and sense organ disorders in children of mothers with obesity compared to children of normal weight mothers is in keeping with those from an Australian study [23] albeit the differences we observed were smaller by comparison. Since specific ICD codes for each visit were not available to us for privacy reasons, we can only speculate which conditions were responsible for the findings. A recent study from Sweden demonstrated an association between maternal BMI and offspring epilepsy [39]; since epilepsy is among the most common neurologic disorders in childhood [40] and its chronic nature results in a higher health care use, this association may have contributed to the observed differences and deserves further investigation. Other potential contributors to maternal obesity-associated morbidity in this ICD chapter may be cerebral palsy or cognitive disorders as previously reported [41, 42]. The higher rates of health care use for respiratory conditions among offspring of mothers with obesity compared to offspring of mothers of normal weight is likely accounted for by higher rates of asthma in the former group [15–17].

This study is one of the first studies to examine the association between maternal pre-pregnancy weight status and offspring health care utilization and cost. We extended the previous literature by using a population-based study design, longer follow-up, and capturing a broad range of primary, secondary, and tertiary level health care use from birth to young adulthood, thereby gaining a broad perspective on the intergenerational effects of maternal pre-pregnancy obesity. This study was strengthened by the universal, single payer health care system in Canada, which reduced selection bias and provided an opportunity to study health care utilization and cost at the population level. Among similar studies, this study has the largest sample size, including over 35,000 children. Our findings should be viewed in context of our limitations. We had to estimate BMI categories for mothers with missing height information from maternal weight based on a more recent sample of women in the database. However, the estimates were highly sensitive and specific and are unlikely to have substantially influenced our findings. Nine percent of births could not be linked with the administrative health data, most likely due to missing or discrepant health card numbers. About 18% of mothers were missing pre-pregnancy weight or their children moved out of the province before 18 years of age and were dropped from analysis. Comparison with the group included in the analysis showed only very minor differences in many maternal characteristics. Pre-pregnancy weight and height were based on self-report at the first prenatal visit, but these data are likely reasonably valid as women gain relatively little weight in the first trimester [43], and self-report can be accurate in situations where women are about to be weighed [44, 45]. If misclassification of weight had occurred, women would have likely underestimated their weight [46], which in turn would have biased regression estimates toward the null as women with overweight and obesity would have been misclassified as normal weight. Paternal weight, which may also be associated with offspring health, was not recorded in our data and, therefore, could not be considered in the analysis. Additionally, we had to estimate the cost per hospital day as hospitals do not operate on a fee-for-service basis but are funded based on patient volume and disease severity. Using the same mean cost per hospital day may have potentially underestimated the difference in costs between offspring of obese and normal weight mothers if the former used hospital resources more intensely due to more complex or severe conditions.

Our cost analyses were limited to the physician and hospital costs and did not include costs for pharmaceuticals, as there is no universal coverage for prescription drugs under the Nova Scotia health care plan. Also, indirect costs (e.g., parent’s lost earnings due to care for a sick child) were not considered. Inclusion of drug costs and indirect costs in the analysis would have likely further increased the cost difference between offspring of mothers with obesity and of normal weight. Another factor that may have contributed to an underestimation of costs is the restriction of the sample to infants without anomalies detected at birth. Since maternal overweight and obesity are associated with an increased risk of congenital anomalies [47, 48], the healthcare utilization and costs found in our study are likely a conservative estimate. Therefore, our findings are only generalizable to infants without anomalies detected at birth.

## Conclusion

Our findings suggest that maternal pre-pregnancy weight status is positively associated with a slightly higher rate of health care use in the offspring. Children of mothers with obesity have approximately $1770 higher direct health care cost over the first 18 years of life and are significantly more likely to be high frequency users of health care than children of normal weight mothers. Respiratory and nervous system disorders contribute most to the difference in health care utilization between the groups. Prevention of maternal pre-pregnancy obesity may not only reduce health care utilization and costs in mothers but also in the offspring. Future research should examine the pathways by which maternal excess weight influences offspring health care use, identify specific conditions that drive those differences, and further characterize high frequency health care users.
